# Sequential anlotinib and camrelizumab combination therapy achieves exceptional survival in multi-driver mutated, TMB-low/PD-L1-low/MSS pulmonary sarcomatoid carcinoma: case report and literature review

**DOI:** 10.3389/fimmu.2025.1718918

**Published:** 2026-01-08

**Authors:** Jun Zhu, Ai Zhu, Gang Li, Lidong Liu, Ziran Gao, Jiayun Liu, Yunfei Ye, Xunzhi Zhu, Yi Li, Hong Chen, Meijin Huang

**Affiliations:** 1 Department of Oncology, 920th Hospital of Joint Logistics Support Force, Kunming, Yunnan, China; 2 Kunming Medical University, Kunming, Yunnan, China; 3 Department of Pathology, 920th Hospital of Joint Logistics Support Force, Kunming, Yunnan, China

**Keywords:** anlotinib, camrelizumab, case report, gene mutation, pulmonary sarcomatoid carcinoma

## Abstract

Pulmonary sarcomatoid carcinoma (PSC) is a rare and aggressive subtype of non-small cell lung cancer (NSCLC) whose molecular characteristics and therapeutic strategies remain poorly defined. This case report documents an exceptional 72-month overall survival in a 40-year-old male patient with stage IVa pulmonary sarcomatoid carcinoma (PSC). The patient harbored seven coexisting driver mutations [*ROS1*, *RET(exon16,exon19)*, *TSC2*, *ALK*, *STK11*, *PTEN*] and exhibited triple-negative immunosuppressive biomarkers: low PD-L1 expression (TPS 3%), low tumor mutational burden (TMB, 11mut/Mb), and microsatellite stable (MSS) status. Sequential anlotinib (anti-angiogenic drug) and camrelizumab (PD-1 inhibitor) combination therapy overcame three biological barriers: (1) angiogenesis inhibition reversed PD-L1 primary resistance by remodeling the tumor microenvironment; (2) treatment induced neoantigens bypassed TMB-L/MSS limitations; (3) multi-target synergy against seven driver mutations. This approach resulted in unprecedented survival outcomes: the 72-month overall survival dramatically exceeds the median OS of less than 12 months reported for advanced PSC, and the patient maintained a progression-free survival of over 37 months on combination therapy, surpassing historical PFS benchmarks. This case provides a clinically actionable framework for managing multi-driver mutated, immunoresistant PSC.

## 1 Introduction

Pulmonary sarcomatoid carcinoma (PSC) is a rare and aggressive subtype of non-small cell lung cancer (NSCLC), constituting merely 0.1%–0.4% of all pulmonary malignancies (1). The prognosis for advanced-stage patients is exceptionally poor, with stage III-IV disease having a 5-year overall survival rate below 5% (2) —significantly inferior to contemporaneous 5-year survival rates in lung adenocarcinoma (20%) (3), squamous cell carcinoma (21.4%) (4), and extensive-stage small cell lung cancer (5.0%) (5), constituting a major therapeutic challenge in oncology. This grave survival reality stems from three interconnected barriers: ① Molecular complexity: The co-occurrence of ≥2 driver gene alterations (e.g., *RET*, *ROS1*, *PTEN*) is observed in up to 32.7% of cases, resulting in objective response rates (ORR) below 15% with targeted therapies (6)^;^ ② Immunosuppressive microenvironment: Only 24% of tumors exhibit PD-L1 expression >1%, coupled with a low mean tumor mutational burden (TMB) of 7.29 mutations/megabase (mut/Mb) (7, 8), collectively confer modest median progression-free survival (PFS) of 5.9–7.2 months with immune checkpoint inhibitor (ICI) monotherapy (9, 10); ③ Clinical guideline restrictions: the NCCN Guidelines (2025 v2) explicitly discourage ICIs in patients with coexisting driver alterations (11), and ESMO consensus warns of an elevated risk of hyperprogression during ICI treatment for sarcomatoid tumors (12). Consequently, PSC remains one of the few solid malignancies in the precision oncology era that lacks standardized therapeutic paradigms, underscoring an urgent need for transformative strategies.

Here, we reported an unprecedented case of stage IVA pulmonary sarcomatoid carcinoma (cT4N3M1a, IVa) achieving 72-month overall survival with sequential anlotinib and camrelizumab combination therapy. Remarkably, the tumor harbored seven coexisting driver mutations (*ROS1*, *RET(exon19)*, *RET(exon16)*, *TSC2*, *ALK*, *STK11*, *PTEN*) and exhibited a “triple-negative” immunosuppressive profile, which we define here as concurrent negativity for the three established predictors of response to immune checkpoint inhibitors: low tumor mutational burden (TMB-Low), low PD-L1 expression(PD-L1-Low), and microsatellite stable (MSS). This profile (TMB-Low, PD-L1-Low, MSS) was confirmed by authoritative assays. The groundbreaking significance of this report lies in its ingenious simultaneous overcoming of three major barriers to treating this type of lung cancer. First, overcoming immunotherapy resistance: The tumor showed primary PD-L1 resistance (PD-L1-Low), making immunotherapy ineffective per NCCN guidelines (2025 v2). Anlotinib (anti-angiogenic drug) remodeled the tumor microenvironment, restoring immunotherapy efficacy. Second, overcoming low immunogenicity: Despite TMB-low and MSS status—features typically linked to poor immunotherapy outcomes—the combination therapy likely enabled effective immune targeting by enhancing recognition of the existing neoantigen repertoire, rather than by generating new neoantigens. Third, defying high-risk mutations: The tumor had 7 concurrent driver mutations, typically fatal (median survival: 10.7 months) (13). The anlotinib plus camrelizumab combo showed strong synergy, extending survival to 72 months (vs. 6 months with immunotherapy alone). This “dual-pronged attack” concurrently disrupted tumor vasculature, activated the immune system, and potentially suppressed resistance mechanisms, extending the patient’s survival to 72 months (6 years). This represents an increase compared to the average survival of less than 6 months for similar advanced-stage patients receiving immunotherapy alone.

To synthesize these observations, a comprehensive literature review was conducted to integrate current evidence on the clinical features, genomic alterations, prognostic factors, and treatment strategies for this rare malignancy. In summary, the camrelizumab-anlotinib combination effectively overcame three critical therapeutic barriers: primary immunotherapy resistance (PD-L1-Low), inadequate tumor immunogenicity (TMB-Low/MSS), and profound oncogenic complexity. This strategy led to an exceptional survival outcome, establishing a novel and clinically actionable paradigm for this refractory subset of lung cancer.

## 2 Case presentation

A 40-year-old male with no significant medical history (including no smoking history) presented on July 15, 2019, with a three-day history of a painless left cervical mass. He reported no associated systemic symptoms (e.g., fever, weight loss) and denied recent infections or trauma. Physical examination​ disclosed a solitary, firm, mobile, mildly tender 2.5 × 2 cm mass in the left supraclavicular fossa/lower cervical chain, without fixation or other lymphadenopathy. Further history-taking revealed no notable family history of cancer, genetic disorders, or relevant psychosocial factors.

**Figure 1:**
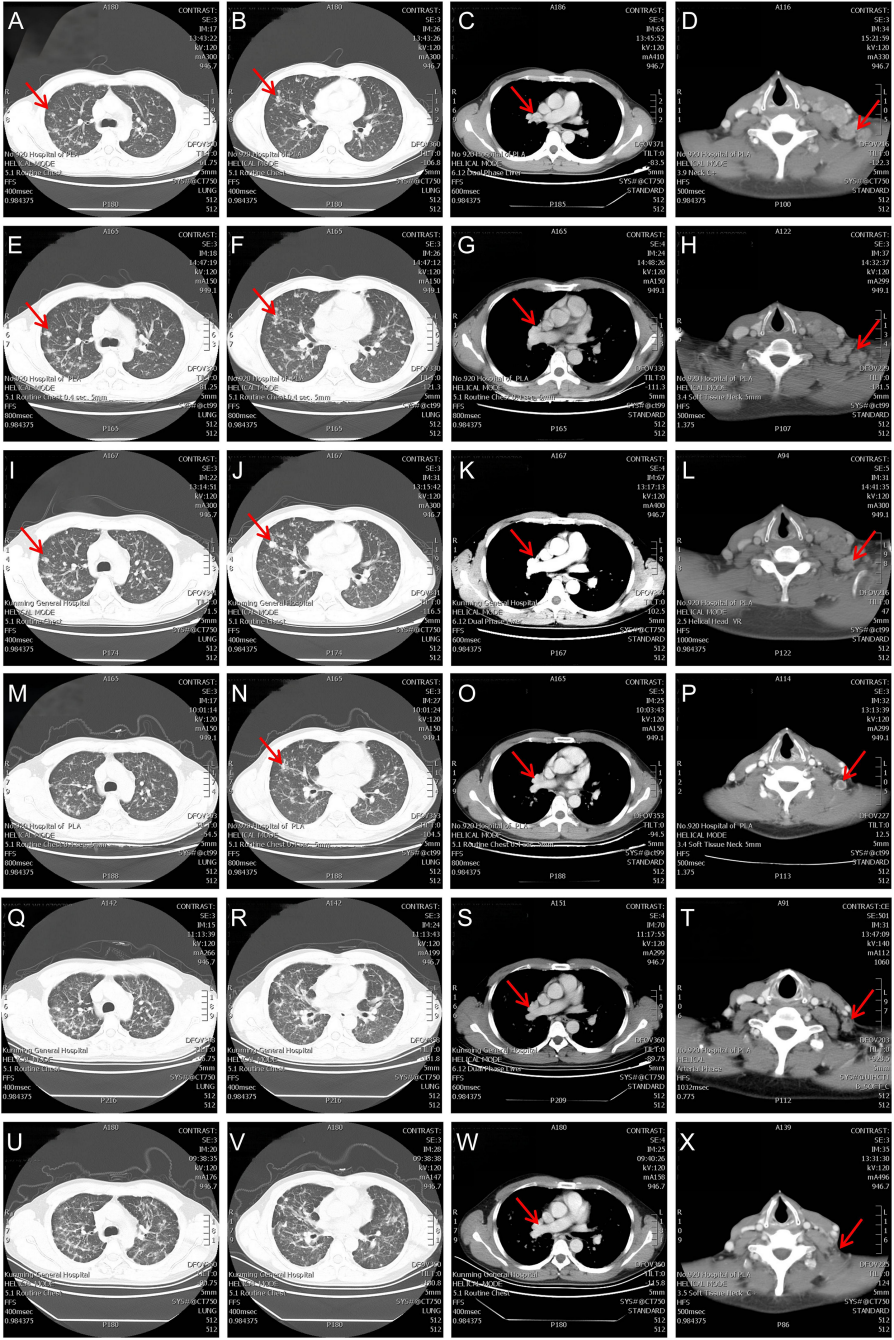
Longitudinal CT evaluation of pulmonary sarcomatoid carcinoma across therapeutic phases. **(A-D)** Baseline (2019-07-17): Left supraclavicular lymphadenopathy (29 mm), right hilar adenopathy (14 mm), and multifocal pulmonary metastases (arrows). **(E-H)** After 2×TP chemotherapy cycles (2021-09-17): Progressive enlargement of bilateral lung metastases (RECIST-defined progression). **(I-L)** After 2×PC chemotherapy cycles (2019-11-11): Continued progression of pulmonary lesions. **(M-P)** After 8×anlotinib cycles (2020-04-07): Significant regression of left supraclavicular nodal and pulmonary metastases. **(Q-T)** Post-31-month anlotinib (2022-06-17): Left supraclavicular nodal progression (PFS1 = 31 months). **(U-X)** After camrelizumab-anlotinib combination (2025-04-28): Stable disease (SD) with sustained remission (PFS2 = 37 months).

On the initial visit, a non-contrast CT of the chest was performed using a Siemens SOMATOM Force 128-slice scanner (July 16, 2019) revealing: 1) Lymphadenopathy: Multiple lymph nodes in bilateral submandibular regions, carotid sheath areas, and left supraclavicular fossa. An enlarged lymph nodes (largest measuring 29mm×19mm) in left supraclavicular fossa, showing suspicious morphology. 2) Pulmonary lesions: Diffusely pulmonary nodules of varying sizes, some with lobulated margins (suggestive of metastatic disease). 3) Pleural changes: Focal pleural thickening with adhesions bilaterally (**Figures 1A–D**). Ultrasonography demonstrated a suspicious 2.6×2.0 cm left neck mass displaying an obscured corticomedullary junction, indicating the need for further evaluation to differentiate between metastasis and lymphoma.

**Figure 2:**
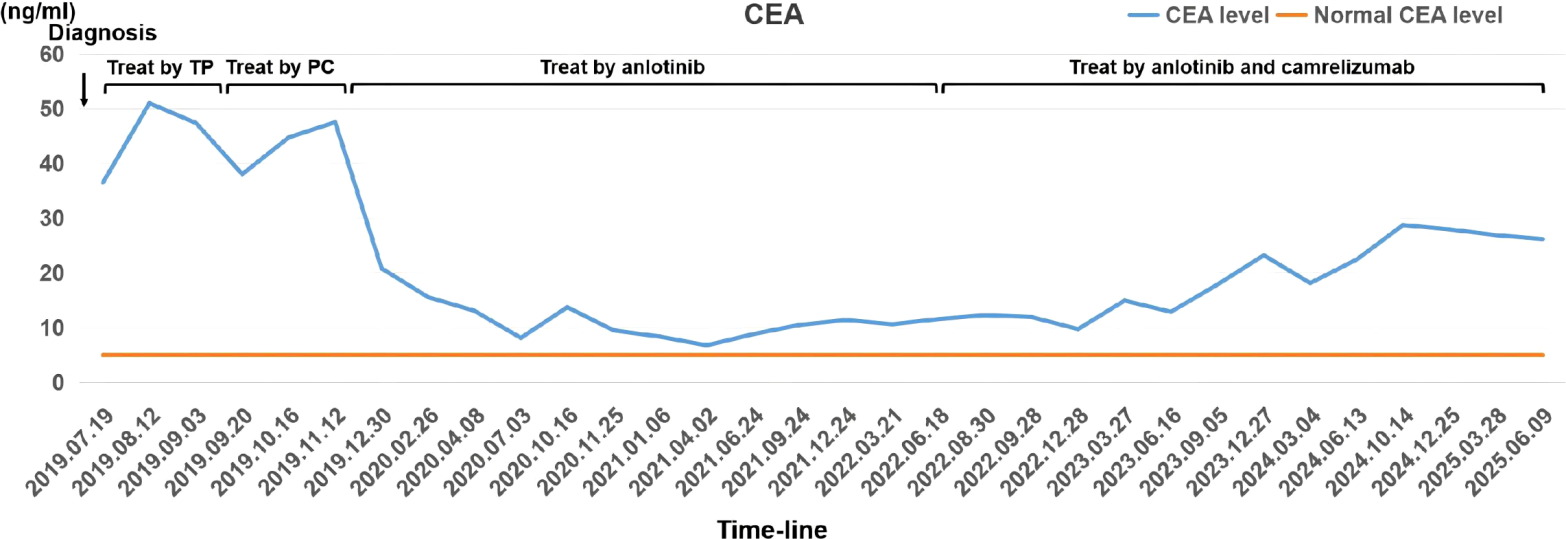
Serial monitoring of serum CEA levels during diagnostic evaluation, treatment course, and clinical follow-up. The plot illustrates CEA concentration dynamics at defined clinical milestones corresponding to therapeutic interventions. CEA quantification was performed by electrochemiluminescence immunoassay (ECLIA) on a Beckman Coulter Access 2 system (Kit REF: A78835; Beckman Coulter, Inc., Brea, CA), with an analytical sensitivity (lower limit of detection, LLoD) of 0.01 ng/mL.

Serum tumor markers were quantified using electrochemiluminescence immunoassay (ECLIA) (Beckman Coulter Access 2) system with manufacturer-matched reagents: Carcinoembryonic antigen (CEA) 36.5 ng/mL (**Figure 2**, **Supplementary Material 1**). The other ten measured tumor markers all demonstrated values within their respective normal reference intervals.

**Figure 3:**
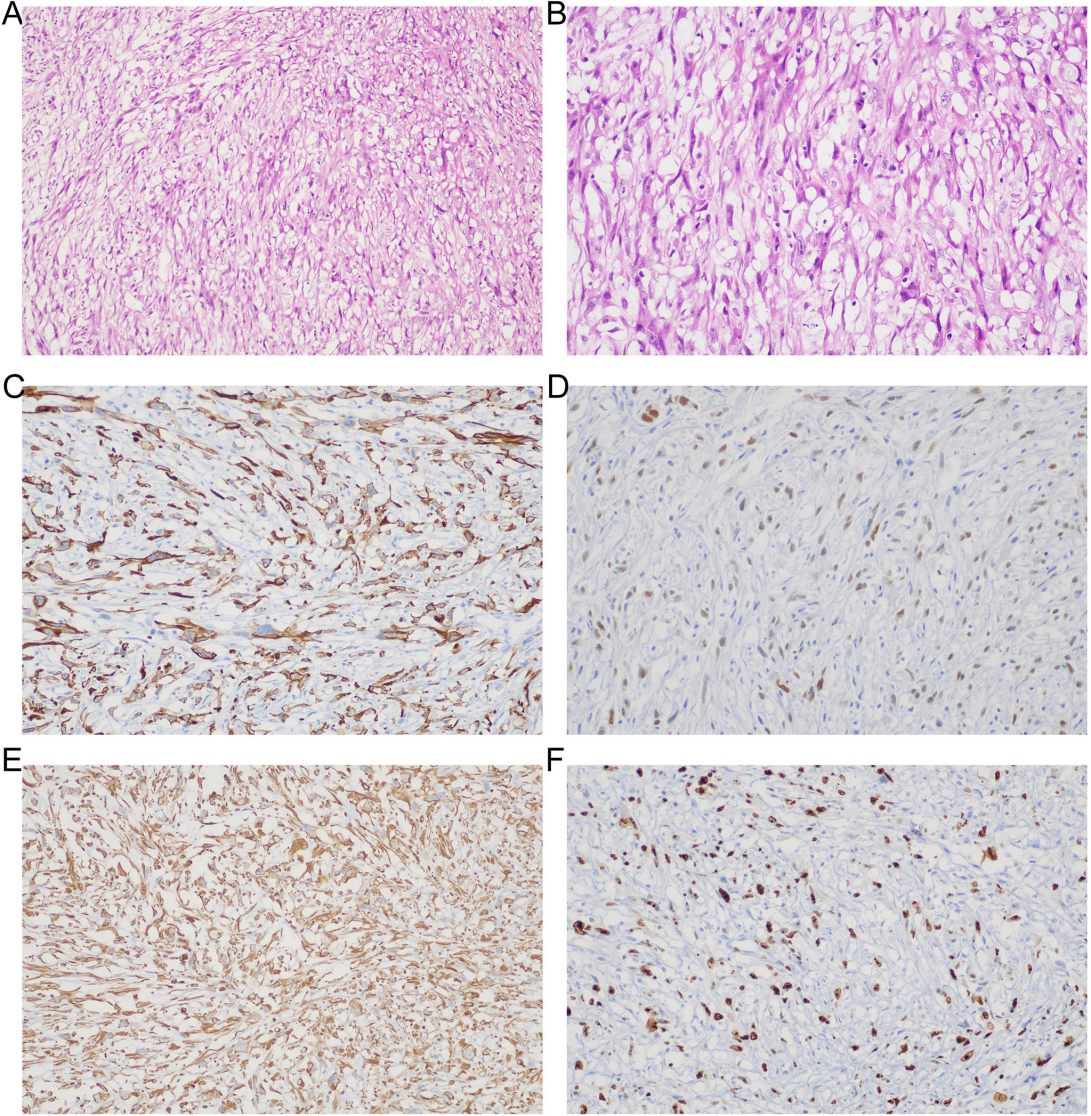
Histopathological and immunohistochemical (IHC) features of left supraclavicular lymph node biopsy. **(A, B)** Hematoxylin and eosin (H&E) staining shows spindle-shaped tumor cells arranged in fascicular architectures at low **(A)**, original magnification ×100) and intermediate **(B)**, original magnification ×200) magnification. **(C)** Immunohistochemical analysis reveals strong diffuse cytoplasmic positivity (2+) for CK7 (original magnification ×200). **(D)** Focal nuclear immunoreactivity (1+) for TTF-1 is observed (original magnification ×200). **(E)** Robust cytoplasmic vimentin expression (2+) is evident (original magnification ×200). **(F)** Ki-67 immunostaining indicates significant proliferative activity (labeling index ≈40%; original magnification ×200).

After timely communication with the patient’s family and consultation with the radiologist, a needle biopsy was recommended for definitive diagnosis. Following exclusion of surgical contraindications and completion of informed consent, a sonographically guided biopsy was performed on the left cervical lymphadenopathy on July 20. Hematoxylin and eosin (H&E) staining revealed spindle-shaped tumor cells arranged in fascicular patterns with marked nuclear atypia, morphologically consistent with the spindle cell subtype of pulmonary sarcomatoid carcinoma. Immunohistochemical analysis demonstrated positive expression of CK7 (++), TTF-1 (+), and Vimentin (++), along with a Ki-67 proliferation index of 40% (**Figure 3**). The tumor was negative for exclusion markers including S-100, CD34, and HMB45. These morphological and immunohistochemical features support the diagnosis of pulmonary sarcomatoid carcinoma.

**Figure 4:**
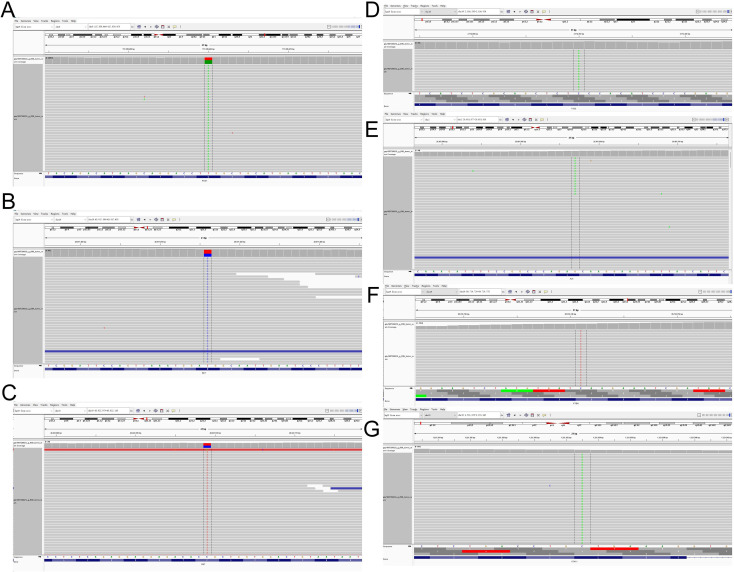
Next-generation sequencing (NGS) analysis of coexisting genomic alterations in pulmonary sarcomatoid carcinoma. **(A)** ROS1 exon31 p.Q1708L (c.5123A>T) with VAF of 59%. **(B)** RET exon19 p.P1039L (c.3116C>T), VAF 46%. **(C)** RET exon16 p.M918T (c.2753T>C), VAF 39%. **(D)** TSC2 exon15 p.S494Y (c.1481C>A), VAF 7%. **(E)** ALK exon16 p.L890F (c.2668C>T), VAF 5%. **(F)** STK11 exon6 p.L286M (c.856C>A), VAF 5%. **(G)** PTEN exon8 p.Q298X (c.892C>T), VAF 3%.

Currently, no standardized treatment protocol exists specifically for pulmonary sarcomatoid carcinoma (PSC). In accordance with the 2018 NCCN Clinical Practice Guidelines for Non-Small Cell Lung Cancer (NSCLC) (14), treatment stratification for advanced NSCLC patients at initial diagnosis should include both genetic testing and PD-L1 expression analysis. Subsequently, postoperative tissue sample of the patient was subjected to targeted next-generation sequencing (NGS) using the NGS-panel 639 (Jiaxin Yunying Pharmaceutical, Jiangsu, China) with matched peripheral blood sequencing for germline variant filtering. Genetic testing revealed: ROS1 exon 31 p.Q1708L (c.5123A>T), with variant allele frequency (VAF) of 59%, RET exon 19 p.P1039L (c.3116C>T) with VAF of 46%, RET exon 16 p.M918T (c.2753T>C) with VAF of 39%, TSC2 exon 15 p.S494Y (c.1481C>A) with VAF of 7%, ALK exon 16 p.L890F (c.2668C>T) with VAF of 5%, STK11 exon 6 p.L286M (c.856C>A) with VAF of 5% and PTEN exon 8 p.Q298X (c.892C>T) with VAF of 3% (**Figure 4**). The patient showed low PD-L1 expression, with a qPCR-derived Ct ratio of 3.08 (19.96/6.48); with tumor mutation burden of 11 muts/Mb and microsatellite low-degree instability type (MSS status, 19.0% STR mutation rate) (**Supplementary Material 2**). Metastatic evaluation comprising neuroimaging (MRI), abdominopelvic CT, and skeletal scintigraphy revealed no extra-thoracic dissemination. Based on imaging, and pathological examination results, in accordance with the ninth edition of the IASLC Lung Cancer TNM staging system, the patient was diagnosed with pulmonary sarcomatoid carcinoma, stage IVa (cT4N3M1a).

The patient had no history of cardiopulmonary disorders, connective tissue diseases, or autoimmune diseases, and had normal hepatic and renal function. The Eastern Cooperative Oncology Group (ECOG) performance status was 1. After a multidisciplinary assessment ruled out contraindications to chemotherapy, the patient received two 3-week cycles of the TP regimen (paclitaxel 220 mg/m² IV on day 1, plus nedaplatin 60 mg/m² IV on days 1–2). After two cycles of chemotherapy, a comprehensive disease evaluation was performed. Chest CT on September 20, 2019, revealed progression of pulmonary lesions, with increased size and number compared to prior imaging (**Figures 1E–H**). Tumor marker analysis showed an elevated CEA level of 38.05 ng/mL. According to RECIST 1.1 criteria, these findings indicate disease progression (PD).

In accordance with guideline recommendations, second-line therapy with the PC regimen (pemetrexed 500 mg/m² IV over 30 minutes on day 1] plus carboplatin [AUC 5 IV over 2 hours on day 1]) was initiated on September 25, 2019, with a 3-week treatment cycle. After two cycles of chemotherapy, the patient underwent comprehensive restaging including imaging and tumor marker evaluation. Radiological evaluation performed on November 13, 2019, documented disease progression, with CT evidence of enlarged cervical lymph nodes and increased extent of pulmonary lesions (**Figures 1I–L**), consistent with clinical deterioration. Tumor marker analysis revealed a rising CEA level to 47.55 ng/mL (**Figure 2**). These findings confirmed disease progression, prompting initiation of third-line therapy with anlotinib (12 mg daily for 14 days followed by a 7-day rest period, constituting a 3-week cycle) starting November 21, 2019. Following the initiation of anlotinib therapy, systematic radiologic and serologic monitoring demonstrated robust treatment efficacy: 1) Radiologic Response (RECIST 1.1 criteria): After 6 cycles, the sum of cervical lymph node diameters decreased by 40.0% (from 25 mm to 15 mm), with dominant pulmonary metastases diameters reduction from 14 mm to 7 mm, meeting criteria for partial response (PR) (**Figures 1M–P**). 2) Tumor Marker Dynamics: Serum CEA levels declined by 72.4% (from 47.55 ng/mL to 13.10 ng/mL). Treatment was well-tolerated with only Grade 1 hypertension (controlled by amlodipine 5 mg daily) and no treatment discontinuations. The patient maintained 100% dose adherence through 32 completed cycles (November 2019–June 2022), representing 31 months of progression-free survival (PFS1).

During the follow-up evaluation on June 20, 2022, neck CT revealed disease progression, characterized by a 50% increase in the short-axis diameter of the left supraclavicular lymph node (from baseline 10 mm to 15 mm) accompanied by elevation of tumor markers (CEA: 6.80 to 11.61 ng/mL) (**Figures 1Q–T**). In response to disease progression, the treatment regimen was modified to combination therapy with camrelizumab (200 mg IV q3w) and anlotinib (12 mg/day, days 1–14 every 3 weeks) starting June 21, 2022.

**Figure 5:**
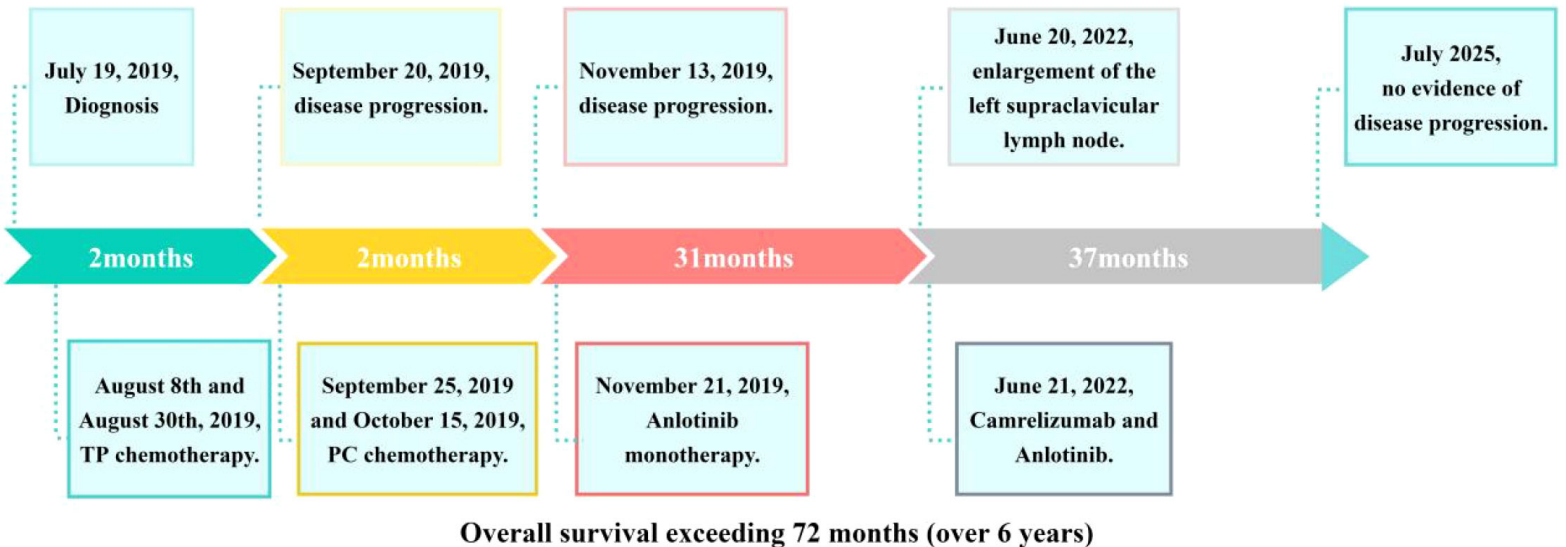
Therapeutic timeline demonstrating sustained disease control in this patient.​.

Through 52 cycles of treatment monitoring (June 2022- July2025), serial radiographic assessments confirmed sustained stable disease (SD) per RECIST 1.1 criteria. Despite fluctuations in serum CEA levels exceeding normal ranges, radiological evaluation confirmed maintained stability of the dominant lesion without significant dimensional changes. The camrelizumab-anlotinib combination maintained favorable tolerability (CTCAE v5.0 Grade 0-1), with no new-onset grade ≥2 adverse events observed throughout treatment. As of July 2025, the patient achieved an exceptional progression-free survival (PFS) of 37 months (**Figures 1U–X**). **Figure 5** presents the comprehensive therapeutic timeline and disease control outcomes for this patient, detailing sequential interventions—including initial chemotherapy, anlotinib monotherapy (PFS1: 31 months), and camrelizumab-anlotinib combination (ongoing PFS2: 37 months)—demonstrating durable disease control with 72-month overall survival and radiographic stability at last assessment (RECIST 1.1).

## 3 Literature review and discussion

### 3.1 Literature review​

A comprehensive literature search was performed across PubMed, CNKI, and other academic databases using the keywords “Pulmonary sarcomatoid carcinoma” to identify pivotal studies and clinical reports related to treatment outcomes and prognosis (**Table 1**).

**Table 1:** Summary of classic clinical studies on pulmonary sarcomatoid carcinoma.

Author	Study Type	Year/Country	Number	Pathologic stage				Treatment methods	PFS (mouths)	Median-OS/survival rate (mouths)
				I	II	III	IV			
Lee J (10)	Retrospective Cohort​	2018/Korea	17	0	0	0	17	Chemotherapy[mesna, doxorubicin, ifosfamide, and dacarbazine (MAID)] (17, 100%)	2.8	Overall 8.7
Davis MP (15)	Retrospective Cohort​	1984/American	17	8	2	6	1	Pulmonary resection/palliative resection/Internal Medicine Treatment(15/1/1, 88.2%/5.9%/5.9%)	——	Overall 12,2-year survival rate 50.0%
Lococo F (16)	Retrospective Cohort​	2017/Italy	148	36	69	33	10	Pneumonectomy/bilobectomy/sublobar resection(8/132/8, 5.4%/89.8%/5.4%)	——	Overall 19, 5-year survival rate 12.6%
Sun L (17)	Retrospective Cohort​	2020/China	175	51	33	60	31	surgical/surgical+neoadjuvant chemotherapy/surgical+djuvant chemotherapy/radiotherapy± chemotherap/best supportive care(53/1/78/32/11, 30.3%/0.6%/44.6%/18.3%/6.3%)	8	Overall 12, 5-year survival rate 25.1%
Steuer CE (18)	SEER database	2017/American	7965	1054	579	1445	2858	Surgery/Radiation/Chemotherapy(3057/3017/2985, 38.4%/37.9%/37.5%)	——	Overall 6.4, stage I-II, III and IV were 16.9, 5.8 and 5.4 months.1-, 3-, and 5-year survival rates were 33.7%, 18.4%, and 14.4%
Xie Y (19)	SEER database	2022/China	400	66	82	91	161	Surgical/ chemotherapy/radiotherapy(197/208/151, 49.3%/52.0%/37.8%)	——	Overall 9,1-, 3-, and 5-year survival rates were 42%, 27%, and 21%
Gong T (20)	Retrospective Cohort​	2022/China	78	29	26	19	4	Surgey/Surgey+radiotherapy ± chemotherapy/Surgey+chemotherapy ± radiotherapy care(78/46/28, 100.0%/59.0%/35.9%)	——	Overall 16.6, 5-year survival rates 3.85%
Abdallah HM (21)	NCDB database	2021/American	1497	678	550	269	0	Adjuvant chemotherapy/no chemotherapy(932/565, 37.8%/62.2%)	——	Stage I, II, III were 56.3%, 41.0%, and 23.1%
Vieira T (22)	Retrospective Cohort​	2013/France	97	0	0	14	83	Platinum-based chemotherapy regimen/non–platinum-based chemotherapy(71/26, 73.2%/26.8%)	2	Overall 6.3
Bae HM (23)	Retrospective Cohort​	2007/Korea	13	0	0	0	13	Chemotherapy	——	Overall 5
Hong JY (24)	Retrospective Cohort​	2008/Korea	12	0	0	0	12	Palliative chemotherapy(12, 100.0%)	——	Overall 8
Tamura Y (25)	Retrospective Cohort​	2015/Japan	16	0	0	2	14	Chemotherapy/chemotherapy+radiotherapy(13/3/, 81.2%/18.8%)	1.5	Overall 7.2
Oizumi S (26)	Phase 2 study(HOT1201/NEJ024)	2022/Japan	16	0	0	0	16	carboplatin/paclitaxel (CP) ± bevacizumab(16, 100.0%)	2.6	Overall 8.8
Lu S (27)	Multicentre, single-arm, open-label, phase 2 study(NCT02897479)	2021/China	25	0	0	1	24	Savolitinib(harbouring MET exon 14 skipping alterations)	5.5	Overall 17.9
Lee J (28)	Retrospective Cohort​	2020/Korea	49	0	0	18	31	Prolizumab/nivolumab/ atezolizumab(40/7/22, 85.1%/14.9%/4.3%)	7.2	Overall 22.2
Inomata M (29)	Multicenter observational study	2022/Japan	22	0	0	0	22	ICI monotherapy/cytotoxic agents + ICI/ICI doublet(15/6/1, 68.2%/27.3%/4.5%)	9.6, 1-, and 5- year PFS rates were 45.5% and 30.1%	1-, and 5- year survival rates were 50.1% and 50.1%
Wu S (30)	Retrospective Case	2023/China	1	0	0	1	0	Pembrolizumab combined with anlotinib(1,100.0%)	——	Overall 45
Qian X (31)	Retrospective Cohort​	2023/China	21	0	0	8	13	Combination therapies(ICIs+anlotinib)/ICIs(13/8, 61.9%/38.1%)	9.2	Overall 22.8
Okauchi S (32)	Retrospective Case	2020/Japan	1	0	0	0	1	pembrolizumab and platinum-containing chemotherapy(1, 100%)	7	——
Kong F (33)	Retrospective Case	2020/China	1	0	0	1	0	camrelizumab plus doxorubicin	20	——
Kim M (34)	Phase II study(KCSG-LU16-07)	2020/Korea	18	0	0	0	18	durvalumab and tremelimumab	5.9	Overall 15.4
Domblides C (35)	Retrospective Cohort​	2020/France	37(4 cases Unknown)	0	4	12	17	platinum based chemotherapy regimen+ICI treatment	Overall 4.89, 1-year PFS rate of 15%	Overall 12.7
Gu L (36)	Retrospective Cohort​	2015/China	95	23	26	30	16	Lobectomy ± radiotherapy ± chemotherapy/total lung resection ± radiotherapy ± chemotherapy/tumor excision ± radiotherapy chemotherapy/chemotherapy ± radiotherapy(81/3/4/7,85.3%/3.2%/4.2%/7.4%)	——	Overall 11.54, 1-, 2-, 3-, and 5-year survival was 32%, 30%, 25%, and 21%
Maneenil K (37)	Retrospective Cohort​	2017/American	127(1 cases Unknown)	20	26	28	52	Surgey ± radiotherapy ± chemotherapy/chemotherapy ± radiotherapy/best supportive care(61/41/25,48.0%/32.3%/19.7%)	——	Overall 9.9,1-, 2-, and 5-year survival rates were 42%, 23%, and 15%
Tamura M (38)	Retrospective Case	2006/Japan	2	0	0	0	2	Surgey/best supportive care(1/1,50.0%/50.0%)	0	Overall 5
Karim NA (39)	Retrospective Cohort​	2017/American	24	5	8	5	6	Surgery/Radiation/Chemotherapy(16/8/7, 66.7%/33.3%/29.2%)	——	Overall 12
Zeng Q (40)	Retrospective Cohort​	2021/China	262	25	70	123	17	Surgery/Surgery+Chemotherapy/neoadjuvant chemotherapy or chemoradiotherapy+ Surgery/Chemotherapy/Radiation/chemoradiotherapy (96/117/26/14/3/6, 36.6%/44.7%/9.9%/5.3%/1.1%/2.3%)	——	Overall 22.0.1-, 3-, and 5-year survival rates were 59.9%, 40.1%, and 36.1%
Liang X (41)	Retrospective Cohort​	2021/China	1723	318	175	427	665	Surgery/Radiation/Chemotherapy(776/685/643, 45.0%/39.8%/37.3%)	——	Overall 8. 1-,2-, 3-, and 5-year survival rates were 38.6%, 26.3%, 22.1% and 18.1%
Sukrithan V (42)	Retrospective Case	2017/American	5	0	0	0	5	pembrolizumab	11+-29+	14+-33+
Li X (43)	Retrospective Case	2018/China	1	0	0	0	1	Apatinib(1, 100%)	14	——
Cimpeanu E (44)	Retrospective Case	2020/American	1	0	0	0	1	Pembrolizumab	14	——
Signorelli D (45)	Retrospective Cohort​	2019/American	16	0	0	0	16	ICIs	2.6	Overall 4.6

Pulmonary sarcomatoid carcinoma (PSC), a rare and highly aggressive subtype of non-small cell lung cancer (NSCLC), is associated with a universally poor prognosis. Historical data underscore persistent therapeutic challenges: Davis et al. (15) first reported a median overall survival (mOS) of 12 months and a 2-year survival of 50% in 17 surgically resected U.S. patients. Subsequent surgical series consistently identified surgery as the cornerstone for localized disease, yet long-term outcomes remained suboptimal. For instance, Lococo et al. (16) observed an mOS of 19 months and a 5-year survival 12.6% in 148 Italian patients (89.8% undergoing lobectomy/bilobectomy), while Sun et al. (17) observed an mOS of 12 months and a 5-year survival rate of 25.1% in 175 Chinese patients (44.6% receiving adjuvant chemotherapy). Large database analyses further highlighted stark stage-dependent disparities. Steuer et al. (18) (SEER *n* = 7965) demonstrated that mOS declined sharply from 16.9 months (stage I-II) to 5.4 months (stage IV), a finding corroborated by Xie et al. (19) (SEER *n* = 400; mOS 9 months, 5-year survival 21%). Even with surgical intervention, outcomes remained dismal—Gong et al. (20) (*n* = 78) reported an mOS of 16.6 months, yet the 5-year survival rate was critically low (3.85%), emphasizing the persistent risk of recurrence. Abdallah et al. (21) (NCDB *n* = 1497) further investigated the stage-specific survival impact of adjuvant chemotherapy, reinforcing the unmet therapeutic needs in this aggressive malignancy.

Conventional chemotherapy yields persistently poor outcomes for advanced or unresectable pulmonary sarcomatoid carcinoma (PSC), with survival outcomes closely aligned with its aggressive tumor biology. Vieira et al. (22) reported a median progression-free survival (PFS) of just 2.0 months and a median overall survival (OS) of 6.3 months in 97 French patients treated with first-line platinum-based or non-platinum regimens. These findings align with multiple palliative chemotherapy studies, including Bae et al. (23) (*n* = 13, mOS 5.0 months), Hong et al. (24) (*n* = 12, mOS 8.0 months), and Tamura et al. (25) (*n* = 16, mPFS 1.5 months, mOS 7.2 months). Similarly, the Oizumi et al. (26) Phase II trial (HOT1201/NEJ024, *n* = 16) evaluating carboplatin/paclitaxel ± bevacizumab showed limited efficacy (mPFS of 2.6 months and mOS of 8.8 months). Notably, Lee et al. (10) investigated a sarcoma-type regimen (MAID; *n* = 17) but found no significant improvement over standard NSCLC chemotherapy (mPFS 2.8 months, mOS 8.7 months), further highlighting the refractory nature of this malignancy.

In recent years, molecularly targeted therapies have transformed the management of cancers driven by actionable genetic alterations. The development of molecularly targeted therapies has introduced new treatment possibilities for specific patient subsets. Lu et al. (27) demonstrated in a phase II trial (NCT02897479) that Savolitinib achieved a median progression-free survival (mPFS) of 5.5 months and a median overall survival (mOS) of 17.9 months in patients with MET exon 14 skipping mutation-positive pulmonary sarcomatoid carcinoma (*n* = 25). This represents a 2.8-fold improvement in mOS compared to conventional chemotherapy (Vieira et al. (22) mOS 6.3 months).

Immune checkpoint inhibitors (ICI) further expand the pool of eligible patients. PD-1/PD-L1 inhibitors have demonstrated survival benefits in individuals with PD-L1-positive tumors. Lee et al. (28) reported an mPFS of 7.2 months and an mOS of 22.2 months in 49 Korean patients, while Inomata et al. (29) (*n* = 22) observed an mPFS of 9.6 months and a 1-year OS rate of 50.1%. Furthermore, combination strategies enhance efficacy through mechanistic synergy. Anti-angiogenic agents (e.g., anlotinib) combined with PD-1 inhibitors reverse the immunosuppressive microenvironment (Wu et al. (30), mOS >45 months; Qian et al. (31), *n* = 21, mPFS 9.2 months/mOS 22.8 months), and ICIs plus chemotherapy (Okauchi et al. (32)^)^ or doxorubicin (Kong et al. (33) PFS 20 months) yield durable responses. However, the efficacy of ICIs exhibits marked heterogeneity: The durvalumab plus tremelimumab regimen (Kim et al. (34),*n* = 18) achieved only an mPFS of only 5.9 months (mOS 15.4 months), and platinum-based chemotherapy plus ICI (Domblides et al. (35), *n* = 37) resulted in a 1-year PFS rate of just 15% (mPFS 4.89 months). These findings underscore the critical need for patient selection based on biomarkers, such as dynamic PD-L1 expression changes and tumor mutational burden (TMB).

In summary, the literature consistently characterizes PSC as a highly aggressive subtype of non-small cell lung cancer (NSCLC). Historical data established surgery as the primary therapeutic approach for localized disease, with median OS typically ranging from 10 to 19 months, but with very low long-term (5-year) survival rates (rarely exceeding 25%). This highlights the high recurrence risk and emphasizes the unmet need for novel therapeutic strategies.

### 3.2 Discussion

This case represents the first report of a stage IVA pulmonary sarcomatoid carcinoma (PSC) patient with seven coexisting driver mutations(*ROS1*, *RET (exon 19)*, *RET (exon 16)*, *TSC2*, *ALK*, *STK11*, and *PTEN*) showing variant allele frequencies (VAFs) ranging from 3% to 59%. Notably, despite an extremely unfavorable tumor microenvironment marked by triple-negative immune resistance markers (PD-L1 TPS 3%, TMB-L 11 muts/Mb, MSS status), sequential therapy with anlotinib and camrelizumab has achieved sustained remission for 72 months—establishing a new survival milestone for this PSC subtype. From a molecular mechanism perspective, the seven driver mutations synergistically drive malignant tumor progression through a complex signaling network. These mutations involve multiple critical signaling pathways, including MAPK/ERK and PI3K/AKT/mTOR, which promote tumor cell proliferation, survival, and invasion via distinct molecular mechanisms (46). For instance, activating mutations in ROS1 and ALK enhance receptor tyrosine kinase (RTK) signaling, while loss of PTEN leads to hyperactivation of the PI3K/AKT/mTOR pathway, further exacerbating the malignant phenotype (47). Additionally, STK11 and TSC2 mutations may confer a growth advantage by dysregulating cellular metabolism and energy homeostasis (48). The synergistic interaction of these mutations not only amplifies cross-activation of signaling pathways but also likely contributes to treatment resistance and increased tumor heterogeneity (49, 50). Particularly, distinct exon mutations in the RET gene (exon 19 and exon 16) may further diversify signaling outputs through allele-specific effects, intensifying tumor aggressiveness and malignancy (51). The coexistence of multiple driver mutations and their networked synergistic effects significantly escalate the tumor’s malignant potential, presenting substantial challenges for clinical management (52, 53).

Furthermore, the clinical implications of the individual mutations warrant careful consideration. While certain variants, such as the RETM918T mutation in exon 16, are well-characterized drivers, the specific pathogenic roles and drug sensitivities of others (e.g., TSC2p.S494Y, STK11p.L286M) may be less definitively established and some could be classified as variants of uncertain significance (VUS). This complex mutational landscape, characterized by high allelic heterogeneity (VAF 3%-59%), likely fostered significant intratumoral diversity and created a formidable barrier to targeted therapies aimed at single oncogenic pathways. In this context, the broad multi-kinase inhibitory profile of anlotinib—targeting VEGFR, FGFR, PDGFR, and c-KIT, among others—may have provided a critical therapeutic advantage. By simultaneously suppressing multiple signaling cascades activated by this co-mutation network, anlotinib potentially achieved a suppressive effect that single-agent targeted therapy could not, thereby mitigating the risk of resistance arising from pre-existing or emergent subclones. This underscores the potential utility of multi-targeted kinase inhibition in managing tumors with complex, co-occurring driver alterations.

This groundbreaking therapeutic case establishes a critical paradigm for precision treatment in pulmonary sarcomatoid carcinoma (PSC), with the 72-month sustained remission significantly overcoming the limitations of traditional therapies. Compared to the median progression-free survival (mPFS) of 1.8-2.0 months reported for platinum-based doublet chemotherapy (TP/PC regimens) [Vieira T et al. (22)], the present case progressed after just 2 cycles of TP regimen (PFS 2 months) and the PC regimen failed after 1.8 months, closely linked to PSC’s unique biological properties. Studies indicate that a sarcomatoid component exceeding 50% significantly increases death and recurrence (54), driven by primary resistance mechanisms: 1) P-glycoprotein (P-gp) overexpression, regulated via the NF-κB pathway (55), which mediates drug efflux synergistically with transporters such as breast cancer resistance protein (BCRP) (56); 2) genomic instability, characterized by early TP53 mutations and late mutations (e.g., ARID2), leading to an elevated TMB (up to 11 muts/Mb) (57, 58); and 3) mutation accumulation and chromosomal rearrangements, which accelerate the evolution of resistant clones. These processes select for treatment-resistant subclones through branching evolutionary patterns, creating a vicious cycle of therapeutic resistance (59, 60). In stark contrast, anlotinib monotherapy achieved sustained remission for 31 months – tripling the mPFS of 9.2 months reported by Qian X et al. (31) – attributable to its dual mechanisms of anti-angiogenesis and immune microenvironment modulation via multi-target inhibition.

Building upon the sustained remission achieved with anlotinib monotherapy, this case demonstrates breakthrough efficacy in the combination immunotherapy setting. The camrelizumab-plus-anlotinib regimen achieved sustained remission exceeding 37 months despite triple immunotherapy resistance markers, significantly surpassing the 7.2-month mPFS of traditional immune checkpoint inhibitors. This response suggests anlotinib may remodel the immune microenvironment through synergistic vascular normalization and epigenetic reprogramming. Specifically, it is hypothesized that the combination therapy unlocked effective anti-tumor immunity by reversing the immunosuppressive microenvironment. Anlotinib, through vascular normalization and modulation of immunosuppressive factors, facilitated enhanced T-cell infiltration and antigen presentation. The subsequent addition of camrelizumab (anti-PD-1) in the anlotinib-primed microenvironment then rescued exhausted T-cells, enabling a potent immune response against tumor cells presenting previously immunologically ignored neoantigens. This supports a model wherein the therapy did not generate *de novo*neoantigens but profoundly enhanced the immunological recognition of the pre-existing neoantigen repertoire, overcoming the constraints of the TMB-Low/MSS phenotype. Research confirms anlotinib, as a multi-target tyrosine kinase inhibitor, exerts dual antitumor actions: 1. Molecularly, it potently suppresses transferrin receptor (TFRC) expression via the VEGFR2/AKT/HIF-1α pathway while inducing durable tumor vascular normalization at low doses, thereby enhancing infiltration of CD4+ T, CD8+ T, and NK cells into the tumor microenvironment (61, 62). 2. Immunologically, it selectively downregulates PD-L1 expression on vascular endothelial cells by inhibiting the PI3K/AKT/mTOR pathway, promoting antigen presentation and reducing immune evasion (70, 71). Multiple studies indicate anlotinib combined with PD-1 inhibitors significantly reduces T-cell exhaustion markers (e.g., PD-1, TIM-3), restoring T-cell function (63, 64). Furthermore, by inhibiting VEGF receptors, anlotinib effectively mitigates PD-1 inhibitor-related cutaneous capillary hyperplasia (RCCEP), improving treatment tolerance and durability (65). This synergy mechanism overcomes the traditional dependence of immunotherapy on high PD-L1, high TMB, or MSI-H status, demonstrating potent tumor burden reduction and improved outcomes. Despite the remarkable efficacy achieved in this case, the potential hemorrhagic risk associated with combining anti-angiogenic agents and immune checkpoint inhibitors requires careful consideration, as documented in recent clinical reports (66). Reassuringly, our patient experienced no significant bleeding events throughout the treatment course—an outcome potentially attributable to anlotinib’s distinct pharmacokinetic profile, its intermittent dosing regimen (12 mg, days 1–14, q3w), and the absence of high-risk tumor features such as cavitation or major vascular invasion. These observations underscore that, while the risk is clinically acknowledged, preemptive assessment and vigilant monitoring are essential to ensuring the safe application of this combination strategy. Previous ctDNA monitoring revealed that anlotinib combination therapy induces reductions in ctDNA abundance that correlate with radiographic response (67), suggesting that early detection of resistant clone expansion (e.g., rising variant allele frequency (VAF) of *PTEN* mutations) via dynamic ctDNA tracking could shift intervention timing from RECIST-defined progression to molecular progression (e.g., VAF>10%). This approach, reported by multiple studies report, extends survival (68, 69), represents a ctDNA-guided innovation that provides a novel paradigm for overcoming resistance. By enabling intervention at the pre-molecular progression stage (prior to radiographic progression), it advances treatment from a “post-radiographic progression” to a “pre-molecular progression” strategy.

Although this single-center observational case lacks a control cohort, direct mechanistic evidence from serial biopsies, and cannot definitively rule out the contribution of the patient’s unique tumor biology to the exceptional outcome,​ it provides significant insights for treating multi-driver mutated, TMB-Low/PD-L1-Low/MSS PSC. We acknowledge that the exceptional response observed may be unique to this patient’s specific biology, and the findings require validation in prospective cohorts. Nonetheless, its clinical relevance manifests in three key aspects: First, it challenges the traditional NCCN guideline (2025.V2) notion that “coexisting driver mutations contraindicate immunotherapy” by providing real-world evidence of potential synergy between antiangiogenic agents and PD-1 inhibitors. Second, it redefines the clinical interpretation of PD-L1 testing (transforming a Ct ratio >3.0 from an indicator of “minimal benefit” into a dynamic monitoring biomarker), introducing the concept of treatment-induced biomarker remodeling. Third, it establishes an early-warning threshold for ctDNA dynamic monitoring, shifting resistance management from passive response to RECIST-defined progression toward proactive intervention. These findings offer novel therapeutic perspectives for the “multi-driver mutated with immunotherapy resistance” subgroup, which accounts for approximately one-third of PSC patients.

### 3.3 Conclusion

This first-reported case of stage IVA pulmonary sarcomatoid carcinoma (PSC) achieved an unprecedented 72-month overall survival with sequential anlotinib and camrelizumab therapy, despite harboring seven coexisting driver mutations and triple immunoresistance (PD-L1-low, TMB-low, MSS). The observed outstanding survival extension challenges current NCCN guideline contraindications for immune checkpoint inhibitors in driver-mutated non-small cell lung cancer. Although the synergistic mechanism between anlotinib-mediated kinase inhibition and camrelizumab-driven immune modulation warrants further validation, this case provides pivotal evidence supporting combination immunotherapy as a viable strategy for molecularly complex, immunoresistant PSC. Future studies should focus on validating integrated biomarker frameworks—incorporating dynamic PD-L1 expression, TMB stratification, and ctDNA monitoring—to guide precision therapy for this aggressive malignancy.

## Data Availability

The original contributions presented in the study are included in the article/**Supplementary Material**. Further inquiries can be directed to the corresponding authors.
